# The value of artificial intelligence for detection and grading of prostate cancer in human prostatectomy specimens: a validation study

**DOI:** 10.1186/s13037-022-00345-6

**Published:** 2022-11-23

**Authors:** Maíra Suzuka Kudo, Vinicius Meneguette Gomes de Souza, Carmen Liane Neubarth Estivallet, Henrique Alves de Amorim, Fernando J. Kim, Katia Ramos Moreira Leite, Matheus Cardoso Moraes

**Affiliations:** 1 Laboratory of Image and Signal Processing of the Institute of Science and Technology of Federal University of São Paulo (Universidade Federal de São Paulo – UNIFESP), Rua Talim 330 Sala 108 - Jardim Aeroporto, São José Dos Campos, SP CEP 12231-280 Brazil; 2 Laboratory of Medical Investigations Number 55 of the Sao Paulo University Medical School – FMUSP, Avenida Dr Arnaldo, 455, sala 2145 – Cerqueira Cesar, Sao Paulo, SP CEP 01246-903 Brazil; 3 Denver Health Medical Center, University of Colorado Anschutz Medical Center, Aurora, CO USA

**Keywords:** Convolutional neural network, Artificial intelligence, Prostate cancer, Pathology, Biopsy image

## Abstract

**Background:**

The Gleason grading system is an important clinical practice for diagnosing prostate cancer in pathology images. However, this analysis results in significant variability among pathologists, hence creating possible negative clinical impacts. Artificial intelligence methods can be an important support for the pathologist, improving Gleason grade classifications. Consequently, our purpose is to construct and evaluate the potential of a Convolutional Neural Network (CNN) to classify Gleason patterns.

**Methods:**

The methodology included 6982 image patches with cancer, extracted from radical prostatectomy specimens previously analyzed by an expert uropathologist. A CNN was constructed to accurately classify the corresponding Gleason. The evaluation was carried out by computing the corresponding 3 classes confusion matrix; thus, calculating the percentage of precision, sensitivity, and specificity, as well as the overall accuracy. Additionally, k-fold three-way cross-validation was performed to enhance evaluation, allowing better interpretation and avoiding possible bias.

**Results:**

The overall accuracy reached 98% for the training and validation stage, and 94% for the test phase. Considering the test samples, the true positive ratio between pathologist and computer method was 85%, 93%, and 96% for specific Gleason patterns. Finally, precision, sensitivity, and specificity reached values up to 97%.

**Conclusion:**

The CNN model presented and evaluated has shown high accuracy for specifically pattern neighbors and critical Gleason patterns. The outcomes are in line and complement others in the literature. The promising results surpassed current inter-pathologist congruence in classical reports, evidencing the potential of this novel technology in daily clinical aspects.

## Background

Prostate cancer is the fifth deadliest cancer in the world and the second most frequent among men. Globocan 2020 data count 1,414,259 new diagnoses and 375,304 deaths in a single year. It is known that asymptomatic early-stage, well treated disease is associated with up to 98% long-term survival according to guidelines [1–3].

Pathological diagnosis still raises divergence between specialists, even despite using the same classical Gleason grading (GG) studies [4–7]. Tumor grading is a cornerstone to guide cancer therapy, raising concerns among practitioners worldwide due to heterogeneity [8–10] and the need for novel tools. GG, described elsewhere [4], is purely visual analytic, throwing the spotlight on the standardization capability of artificial intelligence, with growing evidence in the deep learning field [11, 12].

Since 1998, Convolutional Neural Network (CNN) has been established and become a popular technique for image classification [13] Hence, different topologies have been constructed and evaluated for many applications. Some groups presented data on high-performance CNN Gleason grading standards classification [13–18]. However, in every context, specific possible limitations may be found in data organization, computational cost, and scope or focus of evaluation. These possible limitations may compromise more accurate interpretations for specific contexts. Consequently, it is still necessary for medical literature publications in this field to complement, strengthen, and support the CNN potential and its continuous evolution for this application. Therefore, the purpose of this study is to construct and evaluate a deep learning model for graduate the relevant Gleason patterns.

## Method

### Hypothesis

This study’s hypothesis is that a CNN system could be efficient to graduate the relevant GG patterns. Accordingly, we managed to merge computing engineering science with high-standard pathological reports to construct, train and evaluate a specific CNN system to classify G3, G4, and G5.

### Design

The study design is divided into two main flow of work, Clinical Actions, and Computational Actions, with corresponding procedures.

#### Clinical actions

The laboratory of medical investigations from the Medical School of the University of Sao Paulo (FMUSP) collected 32 previously reported radical prostatectomy specimens. They were colored by hematoxylin and eosin (H&E) method, and scanned by an Aperio® microscope, the slide’s images were analyzed, and Gleason patterns 3, 4, and 5 were delineated by the corresponding specialists. Additionally, images from “the Prostate Cancer Grade Assessment (PANDA) Challenge” were also added to the dataset. Hence, providing a richer dataset to support to the model to improve performance, alongside robustness and capacity of generalizing. PANDA includes two open-access datasets: Karolinska Institute (images divided into background, benign and cancerous tissue) and Radboud University Medical Center (13 images divided into background, stroma, benign tissue, and Gleason patterns 3, 4, and 5). To ensure methodological similarity, only Radboud images were used to improve the initial training sample. All the samples underwent the same screening process previously presented [19].

#### Computational actions

**Fig. 1:**
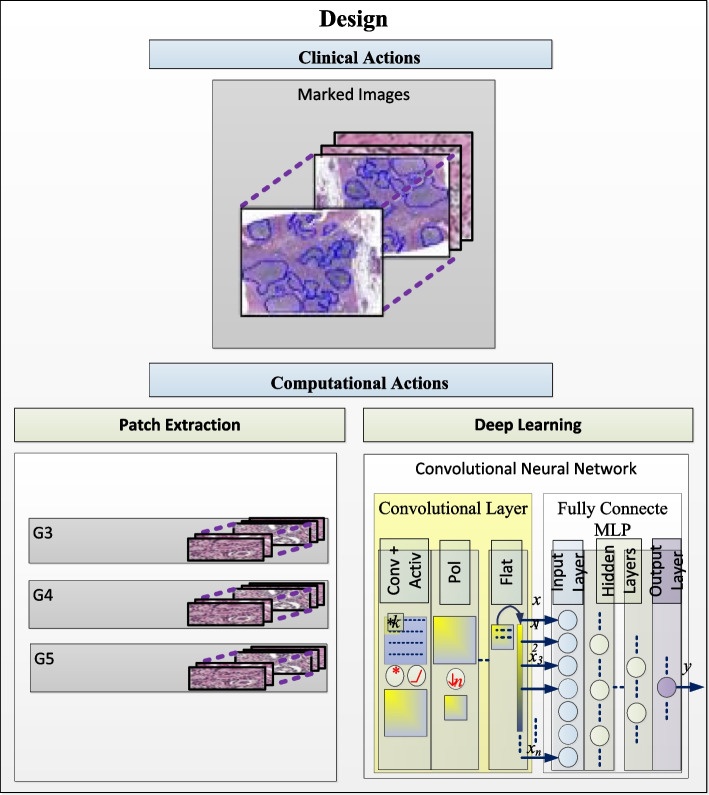
The steps of the Design with their corresponding illustrations: 1^st^ Clinical Actions, resulting in the marked images. 2^nd^ the computational actions showing the two main steps: Patch Extraction Step, and Deep Learning Step

The computational procedures are composed by *Patch Extraction Step, Deep Learning Step* (Fig. 1)*.*

##### Patch extraction step

**Fig. 2:**
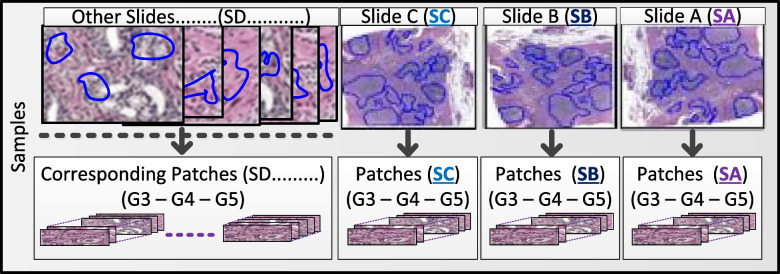
Illustration of patch extraction, connected to its corresponding slides and Gleason grade. Specifically, patches from slides SA, SB, and SC were separated to be applied to the cross-validation process, whereby they were alternately used as training, validation, and test

This step consists in building a dataset of extracted patches, small sample images sized 256 × 256 pixels, with a corresponding 20 × zoom of previously marked regions. The zoom and patch size values were chosen considering they are adequate for individual and combined clinical element identification. Considering this parameter, we obtained a total of 6982 patches (5036 from FMUSP prostatectomy samples and 1946 from the PANDAS Challenge dataset). As a result, patches of Gleason´s 3, 4, and 5 were obtained and can be identified by their corresponding Slide (Fig. 2).

##### Deep learning step

**Fig. 3:**
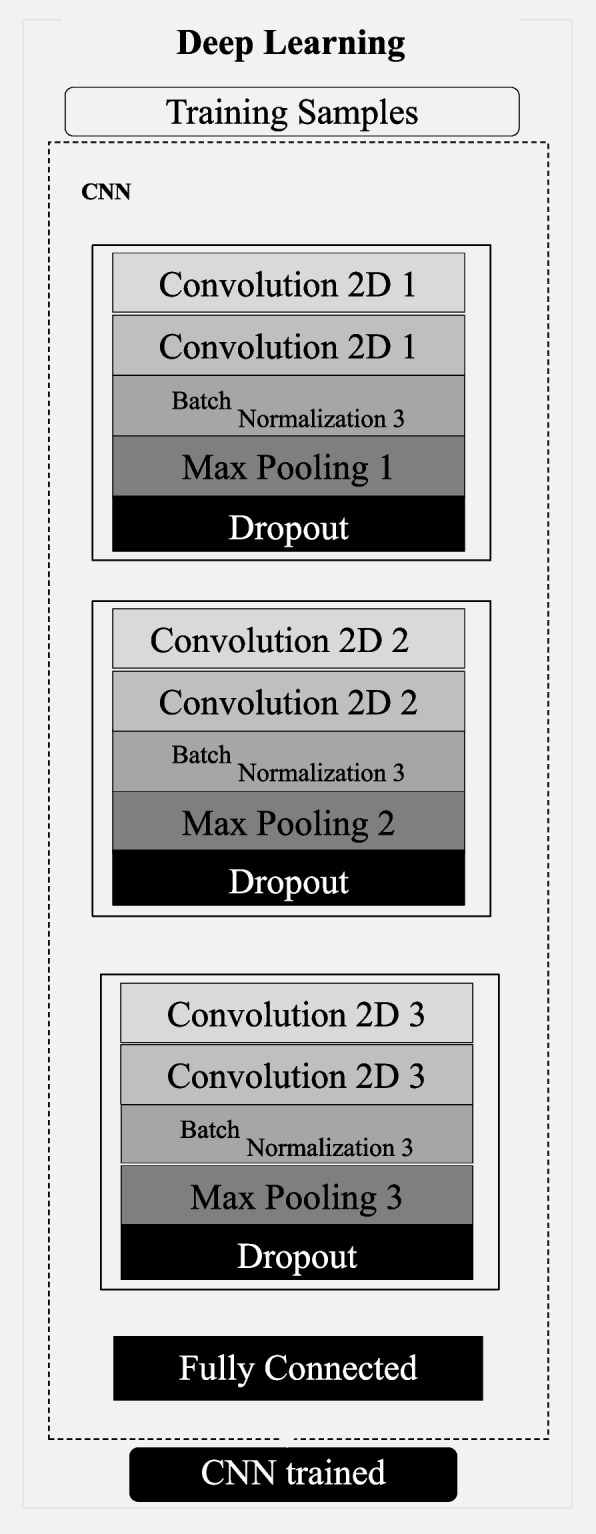
Proposed CNN architecture configuration

This step included topology construction considering a combination of multiple blocks. Previous architectures, characteristics, and important elements were considered to establish the structure proposed. Several experiments were performed using features of complex neural nets, combined blocks, and learning methods, resulting in the obtention of a high-performing architecture for this purpose, as shown in Fig. 3. The neural net input starts with two convolutional layers containing 32 filters with 5 × 5 kernel, and 64 filters with 5 × 5 kernel, respectively. The number of filters is related to the feature extraction diversity in the input – the more filters, the more complementary features are extracted and considered to support decision. The batch normalization layer standardizes output values of the corresponding layer, decreasing the chances of value range saturation. Max Pooling decreased the feature matrix dimension, allowing only the best parameters to proceed; the first sequence ends in a dropout layer. The other sequences (Second and Third) work similarly, except for the number of the filter (64 and 128 in the second, 128 and 256 in the third). Lastly, information goes through the Fully Connected layer containing 512 neurons within the hidden layer, batch normalization; the dropout of 0.5. SoftMax was used as an activation function. RMSprop was chosen as an optimizer for training; thus, the neural net can output the images classified as Gleason patterns 3, 4, and 5.

**Fig. 4:**
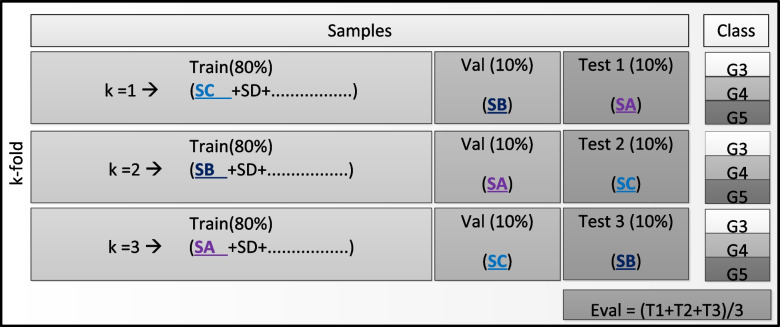
Illustration of how the image slides were separated for the cross-validation process, alternating slides SA, SB, and SC to be used as the source of patches for validation, test, and completing the rest of the training data, hence generating the final classification according to Gleason grades (G3, G4, and G5)

The patch images extracted (Fig. 2) were applied to the architecture described (Fig. 3) for training and evaluation. The separation of training, validation, and test groups was performed using the 80%, 10%, and 10% ratios. In addition, to obtain the most from our image set, we carried out a 3-time k-fold cross-validation, as shown in Fig. 4.

Specifically, this cross-validation took patches from slides SA, SB, and SC (Figs. 2 and 4) to be individually used as validation and test, and the patches from the spare slides complete the training data. This prevented patches of the same patient and slides from being present in the training, validation, and test groups; accordingly, providing a wider context variation, and composed outcome, thus leading to a more reliable and unbiased outcome, supporting better interpretation for corroboration. Slides SA, SB, and SC were chosen because each of them had the most balanced distribution of patches for Gleason 3, 4, and 5. The corresponding distribution and number of patches used for each k of the k-fold can be seen in Table 1. Finally, to improve model accuracy together with robustness, minimizing potential overfitting, data augmentation was performed before being applied to the neural net; specifically, this process includes random rotations, brightness, and zoon.

**Table 1:** Dataset separation considering, approximately, 80%, 10%, 10% ratio, for training, validation, and test of each corresponding k, respectively

*k-fold*	*Class*	*Train(n)* ~ *80%*	*Val(n)* ~ *10%*	*Test(n)* ~ *10%*
*k* = *1*	*Slides*	*SC* + *SD* + ***…***	*SB*	*SA*
	*Gleason 3*	614	73	69
	*Gleason 4*	2205	271	258
	*Gleason 5*	2801	348	349
*k* = *2*	*Slides*	*SB* + *SD* + ***…***	*SA*	*SC*
	*Gleason 3*	*593*	*69*	*94*
	*Gleason 4*	2224	259	251
	*Gleason 5*	2821	352	325
*k* = *3*	*Slides*	*SA* + *SD* + ***…***	*SC*	*SB*
	*Gleason 3*	*589*	*94*	*73*
	*Gleason 4*	2185	281	268
	*Gleason 5*	2807	339	352

The evaluation process occurred in two steps for each k during the proposed k-fold cross-validation (Fig. 4). The first was the training and validation step, and the second was the test, computing typical and additional parameters of performance for better interpretation. The training and validation step evaluate the potential of learning the current application; thus, the parameters accuracy and loss were computed to validate this step. Once high rate of accuracy and loss were achieved, the corresponding trained CNN topology were saved and submitted to the test step. The test step corroborates the previously obtained accuracy; as well as, measuring robustness and potential of generalize classification. During the test step, the corresponding image samples were applied to the trained CNN topology to be classified. As a result, generating the regarding confusion matrix; hence, allowing computing precision, sensitivity, and specificity, for interpretation of possible consequences.

## Results

### Training and validation step

**Fig. 5:**
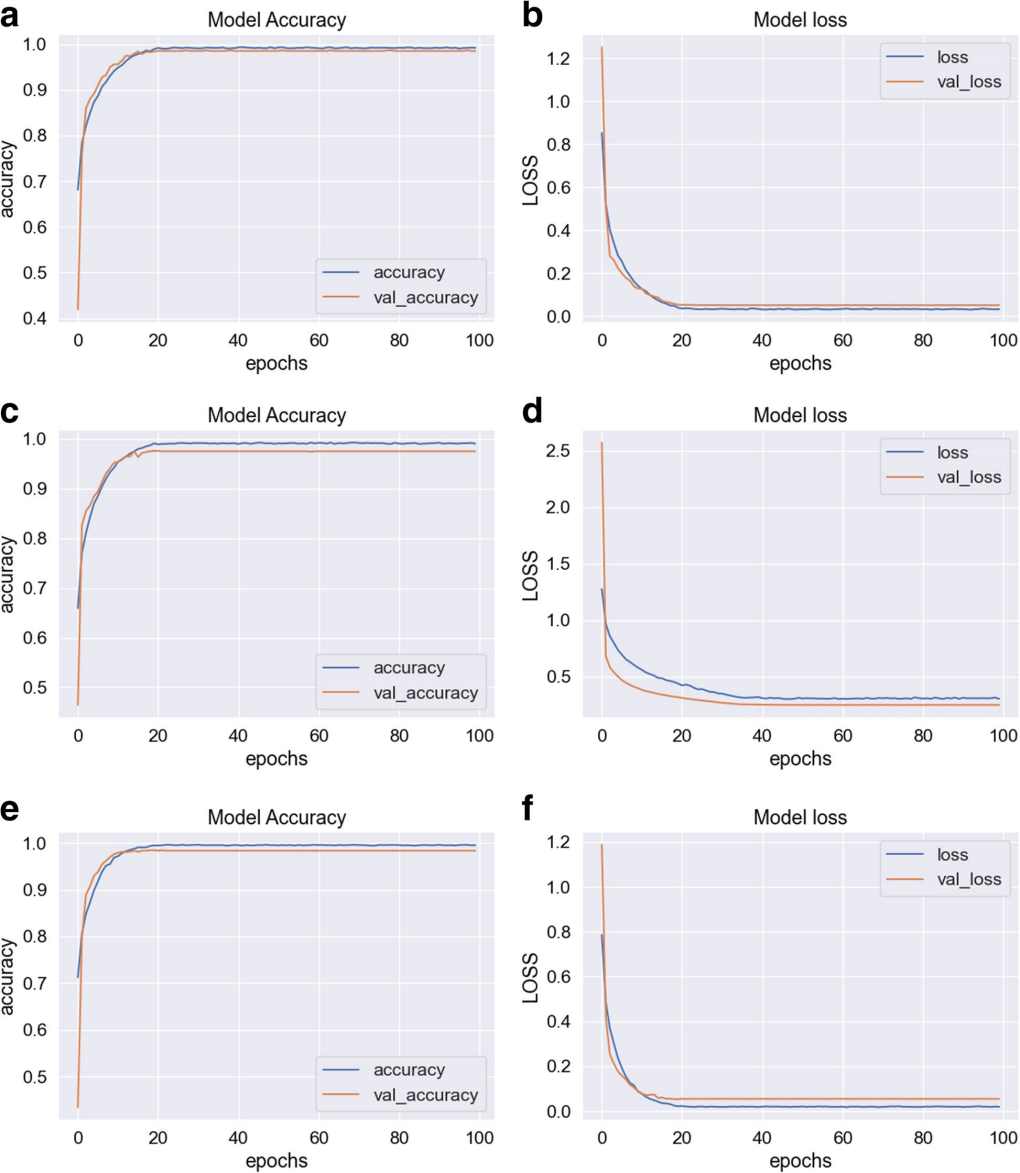
Training and validation curves for each of the three kfold-cross-validation, the blue curve represents training patches and the orange curve represents validations patches. **a** Accuracy for the first cross-validation. **b** Loss for the first cross-validation. **c** Accuracy for the second cross-validation. **d** Loss for the second cross-validation. **e** Accuracy for the third cross-validation. **f** Loss for the third cross-validation

As can be seen in Fig. 5, among the different groups of the three k-folds, the training and validation curves were convergent in terms of accuracy and loss. For training and validation, the accuracy reached about 98%, and loss variates around 1% to 2.5%. In addition, the differences shown in Fig. 5 demonstrate there is no considerable underfitting or overfitting. Considering the proposed context, using slides from different patients for training and validation, in addition to the two different image sources to train the topology, the learning potential of the network is demonstrated. The total training processing time was estimated at 1200 s using one of our laboratory computers (Intel Core i7 3.50 GHz configurations, NVIDIA GeForce GTX 1060 Graphics, 16 GB RAM, 2 TB Hard Disk).

### Test step

**Fig. 6:**
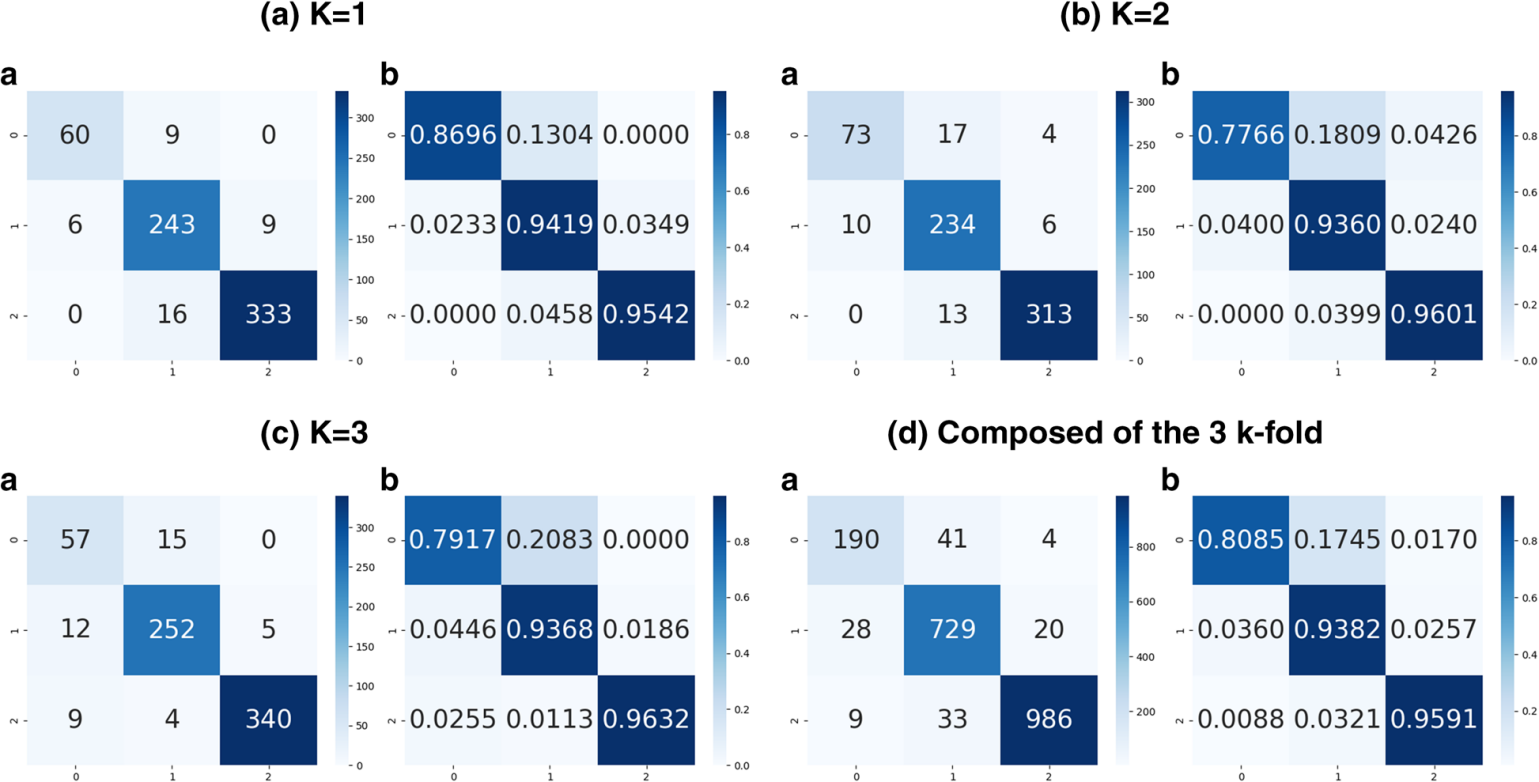
Resulting confusion matrix of the corresponding set of *test* patches. **a**, **b**, and **c** Confusion matrixes of k = 1, 2, and 3, respectively. **d** Confusion matrix with the composed result of the 3 k-folds

After the training and validation of each k, the corresponding trained model was subjected to the test step (Fig. 4). The test step was carried out using the corresponding test data of each cross-validation (Table 1). For each k, the corresponding test data had never been seen by the model; hence, the network was blinded for every set of the test image to prevent bias. The confusion matrix with the results of each k-fold analysis group is presented in Figs. 6a, b, and c; additionally, the composition of all k-fold results is presented in Fig. 6d. From the resulting confusion matrixes, we obtained the pertinent metrics of efficacy, in addition to the general accuracy; precision, sensitivity, and specificity were also computed considering each class as a target object, therefore measuring the potential performance of the model to separate each class. Table 2 summarizes the findings with accuracy, precision, sensitivity, and specificity. Accuracy of around 95% was achieved in the final evaluation data from tests results (Table 2). Additionally, values above 80% and close to 98% were achieved for precision, sensitivity, and specificity for the different classes of Gleasons (Table 2). Considering the blind test applied, the classification potential of the network was evidenced.

**Table 2:** Precision, Sensitivity, Specificity, and Accuracy for each of the corresponding k

*k-fold*	*classe*	*Precision(%)*	*Sensitivity (%)*	*Eespecificity (%)*	*Accuracy(%)*
*k* = *1*	*Gleason 3*	90.91	86.95	99.01	94
	*Gleason 4*	90.67	94.18	94.02	
	*Gleason 5*	97.36	95.41	97.25	
*k* = *2*	*Gleason 3*	*87.95*	*77.65*	*98.26*	95.02
	*Gleason 4*	88.63	93.6	92.85	
	*Gleason 5*	96.9	96.01	97.09	
*k* = *3*	*Gleason 3*	*73.07*	*79.16*	*96.62*	95.67
	*Gleason 4*	92.98	93.68	95.52	
	*Gleason 5*	98.55	96.31	98.53	
*média geral*	***Gleason 3***	***83.7***	***80.85***	***97.95***	**94.89**
	***Gleason 4***	**90.78**	**93.82**	**94.14**	
	***Gleason 5***	**97.62**	**95.91**	**97.62**	

Specifically, the lowest performance values for Gleason 3, compared to patterns 4 and 5 (approximately 81% and 87%, for precision and sensitivity), can be explained by the lower number of samples for this class. Considering the way the patches were obtained, Gleason 3 is lower grade, more similar to noncancer tissue, and has lower volume in prostate specimens resulting in less material for analytical purposes. The consequences of this difference in the number of samples can also be seen with the lower specificity of the other two classes compared to Gleason 3, see Table 2. Considering the clinical relevance of patterns 4 and 5 and eventual non-relevant pattern 3 findings, this disadvantage may represent minimal clinical relevance.

## Discussion

Inter-pathologist grading discordance is known to be a relevant issue in prostate cancer treatment with numerous clinical consequences. Classical studies show inter-pathologists concordance varies between 51–78%, with a greater effect on patterns 3 and 4 differentiation [8–10]. Artificial intelligence (AI) is growing in importance among novel technologies in diagnostic procedures, mainly involving pattern recognition, being widely used in pathology and radiology [20–22].

Multiple recent literature reports on prostate cancer pathology and AI usage show the relevance of this theme [13, 14, 23, 24]. In a recent paper from the Karolinska Institute, Ström et. al. [23], the authors obtained 96–99% accuracy in terms of benign-malignant differentiation and Gleason Grade concordance kappa of 0.62. Bulten et al. [25], used a dataset composed of 5759 prostate biopsies and reached a kappa agreement of 91.8% with pathologist reports. Patches were used with sufficient zoom to find fundamental structures and to classify samples between cancer and non-cancer as well as to provide GG differentiation. Tolkach et al. [17] have separated cancer and non-cancer (stroma, glandular, and non-glandular benign tissue) patches obtaining more than 1.67 million patches.

The studies above have greatly contributed to the current knowledge of AI application to this field. Nonetheless, investigations and alternative approaches with new topologies must be continuously carried out and constructed to complement the current knowledge. We have thus developed a topology with tuned parameters regarding the number of filters, kernel sizes, layer sequence, number of hidden layers, activation function, and optimizer. Our proposed investigation and implementation complement and support the studies carried out in different contexts. The promising results, showing the performance of grading G3, G4, and G5, are in line with the literature, hence reinforcing the high potential of AI methods for this classification. Additionally, alternatives are offered to be used and evolved, contributing to the growing knowledge and evidence in this field.

As a limitation, the limited number of samples is noted for Gleason 3 pattern. However, Fig. 6 demonstrates that misclassification between classes in terms of numbers and percentages still statistically motivating. Furthermore, most misinterpretations are between neighboring Grades 3 and 4 (only 1 patch of Gleason 3 was interpreted as Gleason 5) with a minimal potential of clinical repercussion. Accordingly, considering that patches represent small portions of a large area, these few misinterpretations have minimal significance for the classification of the whole pathological area of the slide, minimizing possible interpretation effects.

Future work will focus on gathering additional collaborators and performing investigations, parallelly evaluating different promising topologies with the same dataset. With a dataset with wider variances, we will obtain the differences among topologies performance. Finally, we will include the construction of a mosaic from classified patches, creating heat map images, and provide a classification of the whole digital slides.

## Conclusion

Pathology is a cornerstone to support intervention discussion between practitioner and patient in actual customized prostate cancer care involving novel therapies (active surveillance, focal) [26, 27] and classic radical ones (radiation therapy and radical prostatectomy) [28]. Artificial intelligence has demonstrated its great potential in helping pathology pattern recognition with high accuracy. Our proposed CNN model added evidence to supports this potential and provides a new alternative to be used and evolved, following the trend towards clinical usage in medical daily practices, consequently increasing the standards on pattern recognition to optimize clinical decisions, enabling best therapeutical results.

## Abbreviations

AI: Artificial Intelligence
CNN: Convolutional Neural Network
GG: Gleason grading scale
H&E: Hematoxylin and eosin
KRML: Initials of the uropathology specialist’s name
PANDA: The Prostate Cancer Grade Assessment
CAPES: Coordination for the Improvement of Higher Education Personnel
FMUSP: University of São Paulo Scholl of Medicine
UNIFESP: Federal University of São Paulo
